# A Scoping Review of Spatial Analysis Approaches Using Health Survey Data in Sub-Saharan Africa

**DOI:** 10.3390/ijerph17093070

**Published:** 2020-04-28

**Authors:** Samuel Manda, Ndamonaonghenda Haushona, Robert Bergquist

**Affiliations:** 1 Biostatistics Research Unit, South African Medical Research Council, Pretoria 0001, South Africa; 2 Department of Statistics, University of Pretoria, Pretoria 0002, South Africa; 3 School of Mathematics, Statistics and Computer Science, University of KwaZulu-Natal, Pietermaritzburg 3209, South Africa; 4 Division of Epidemiology and Biostatistics, University of Stellenbosch, Cape Town 8000, South Africa; 5 Ingerod, SE-454 94 Brastad, Sweden

**Keywords:** spatial methods, disease mapping, health surveys, Sub-Saharan Africa

## Abstract

Spatial analysis has become an increasingly used analytic approach to describe and analyze spatial characteristics of disease burden, but the depth and coverage of its usage for health surveys data in Sub-Saharan Africa are not well known. The objective of this scoping review was to conduct an evaluation of studies using spatial statistics approaches for national health survey data in the SSA region. An organized literature search for studies related to spatial statistics and national health surveys was conducted through PMC, PubMed/Medline, Scopus, NLM Catalog, and Science Direct electronic databases. Of the 4,193 unique articles identified, 153 were included in the final review. Spatial smoothing and prediction methods were predominant (n = 108), followed by spatial description aggregation (n = 25), and spatial autocorrelation and clustering (n = 19). Bayesian statistics methods and lattice data modelling were predominant (n = 108). Most studies focused on malaria and fever (n = 47) followed by health services coverage (n = 38). Only fifteen studies employed nonstandard spatial analyses (e.g., spatial model assessment, joint spatial modelling, accounting for survey design). We recommend that for future spatial analysis using health survey data in the SSA region, there must be an improve recognition and awareness of the potential dangers of a naïve application of spatial statistical methods. We also recommend a wide range of applications using big health data and the future of data science for health systems to monitor and evaluate impacts that are not well understood at local levels.

## 1. Introduction

Spatial analysis concerns the use of statistical methods to analyze spatial data by accounting for location-specific information, elevation, distance, spatial relationships and association between the data [1,2]. These methods are prominent statistical tools in the health and epidemiological sciences where the study of the impact of geographical distribution with respect to health data and outcomes is a major research undertaking. For example, the analysis may identify areas of elevated risk of a disease incidence and prevalence. Such a finding could generate scientific questions and hypotheses about the disease aetiology or provide enough supporting scientific evidence to guide public health recommendations on the disease and geography.

In the context of the United Nation’s sustainable development goals (SDGs) to be achieved by 2030 [3], those related to ending poverty, terminating malnutrition and improving health in general are of interest here. A focus across the SDG goals and targets is on monitoring progress at the sub-national level to avoid national-level statistics masking local heterogeneities. Increased focus on sub-national assessments, efficient targeting of resources and improved accuracy for health and development metrics have prompted an emphasis on the development of spatial analyses to provide estimates at lower national levels [4,5,6]. To meet the need of supporting local-level policies, the implementation and application of spatial techniques have grown exponentially in recent times. This has been made possible by a rise in the availability of nationally representative household and health survey data and high-performance computers to fit spatial statistics methods. Classic spatial statistics methods can now be fitted to larger and more complex spatial datasets in several spatial analysis computer software programs such as SaTScan [7], GeoDa [8] and ArcGIS [9]. Even Bayesian spatial inference, which was intractable before, is now routinely being used to analyze complex spatial models and datasets. Bayesian approaches rely on increased access to spatial statistics software, for example, BayesX [10], WinBUGS/OpenBUGS [11] and Integrated Nested Laplace Approximations (INLA) [12], all freely available applications.

On the other hand, health surveys such as demographic and health surveys (DHS), Malaria Indicator Surveys (MIS), AIDs Indicator Surveys (AIS) and Multiple Indicator Cluster Surveys (MICS) cover a wide range of health topics. Analyses of data from nationally representative households and population health surveys have been done and the findings have provided enough evidence to track the progress of health and socio-demographic indicators to meet local, national and international goals. Even though these surveys are implemented at comparatively enormous costs, their usage has remained sub-optimal since such analyses demand advanced data management and often complicated statistical techniques [13]. A comprehensive analysis using appropriate spatial statistical methods can provide appropriate supporting scientific evidence to guide policy recommendations on health disparities and place.

Even though the application of spatial statistics to map health outcomes and processes have grown in Sub-Saharan Africa (SSA) over the past two decades, reviews summarizing a body of research studies that have employed spatial analysis methods based on nationally representative health survey data are scarce. One previous review on spatial analysis methods on health issues in Africa only applied to HIV research and was general in its coverage of data sources [14]. We set out to review all published literature that employed spatial analysis techniques to nationally representative health survey data in the SSA region. An identification and a description of the spatial analysis methods, software and health discipline used in the applications of spatial statistics to health survey data would be useful to health science researchers including spatial statisticians. We also wanted to identify knowledge gaps and provide useful recommendations for carrying out improved spatial analysis using health survey data in the SSA region. A useful methodology for qualitatively exploring the content of literature through concepts and thematic mapping is conducted using scoping, as opposed to systematic, reviews [15].

## 2. Methods

### 2.1. Eligibility Criteria

Inclusion criteria: articles published in English during the period 1990–2018 employing spatial statistic methods in the SSA region to analyze nationally representative household and health survey data.

Exclusion criteria: articles published outside the 1990–2018 period and all publications based on data from health surveys conducted outside the SSA region, systematic reviews and meta-analyses, publications that only referenced health surveys but did not analyze the data obtained, studies that used non-nationally representative local or regional health surveys data and those that had utilized non-spatial statistical methods such as multilevel/random-effects models. Spatial analyses that used surveillance data were also excluded.

### 2.2. Search Methods

We conducted this scoping review according to the Preferred Reporting Items for Systematic reviews and Meta-Analyses (PRISMA) extension for Scoping Reviews (PRISMA-ScR) guidelines [16]. A Checklist for Preferred Reporting Items for Systematic reviews and Meta-Analyses extension for Scoping Reviews (PRISMA-ScR) is provided as supplementary material (Table S1). However, it has no published protocol. An organized literature search for articles that applied spatial statistical methods and that were published from 1990 to 2018 using data from household and population health surveys was done through PubMed Central (PMC), PubMed/Medline, Scopus, NLM Catalog, and Science Direct electronic databases. Three different searches were conducted for the three NLM literature resources (PMC, PubMed/Medline and NLM Catalog). Our search strategy was formulated using the following keywords to broaden the retrieval of relevant articles: spatial statistics; spatial modelling; spatial variation; small areas estimation; demographic and health survey; AIDS indicator survey; malaria indicator survey; multiple indicator cluster survey; health survey; Sub-Saharan Africa. The search strategy was built using Boolean operators “AND/OR” with keyword combinations, e.g., “spatial statistics” OR “spatial modelling” OR “spatial variation” OR “small areas estimation” OR “demographic health survey” OR “AIDS indicator survey” OR “malaria Indicator survey” OR “multiple indicator cluster survey” OR “health survey” OR “MIS” AND “sub-Saharan Africa”. Correspondingly, filters were applied to restrict our search to the inclusion criteria. A rigorous search of the Cochrane library was done to confirm whether there were existing or ongoing systematic reviews related to this review.

### 2.3. Study Selection

All potential studies retrieved were first imported to Mendeley and duplicates were removed. The remaining articles were imported to Covidence, a web-based systematic review software-designed process of screening, data extraction and analysis [17] for screening. Using the pre-specified inclusion criteria, the article’s titles and abstracts were screened by two independent reviewers. Articles deemed irrelevant were removed during the screening of abstracts and titles. For articles that could not be clearly depicted as relevant or irrelevant during the screening of abstracts and titles, their full-text articles were retrieved for further scrutiny. Full-text articles meeting the inclusion criteria were assessed further, and the following information answering the review’s objectives were abstracted from each paper: spatial statistical method and computer software packages used; data source; health discipline and themes; demographic group studied; and study country or countries. Discrepancies from independent reviewers were resolved through a discussion.

### 2.4. Data Extraction

Data extraction was performed using Microsoft Excel, which produced a master table with the following information extracted from each paper: spatial statistical methods and software; data source; public health outcomes and themes; and demographic focus groups. Spatial analysis techniques were categorized as spatial descriptive or aggregation method; spatial autocorrelation and clustering; spatial regression and interpolation and spatial modeling and prediction. The categories for health disciplines and themes were health service coverage; mortality; malaria and fever; diarrhea; malnutrition; non-communicable diseases; TB and HIV/AIDS; and others. Articles were permitted to be sorted into more than one methodological class and public health themes deemed appropriate. Counts and proportions were primarily used to summarize the study findings. The demographic focus groups were categorized into children (<15 years old) or adults (≥15 years of age) and gender. The study quality was not assessed.

## 3. Results

### 3.1. Study Characteristics

**Figure 1:**
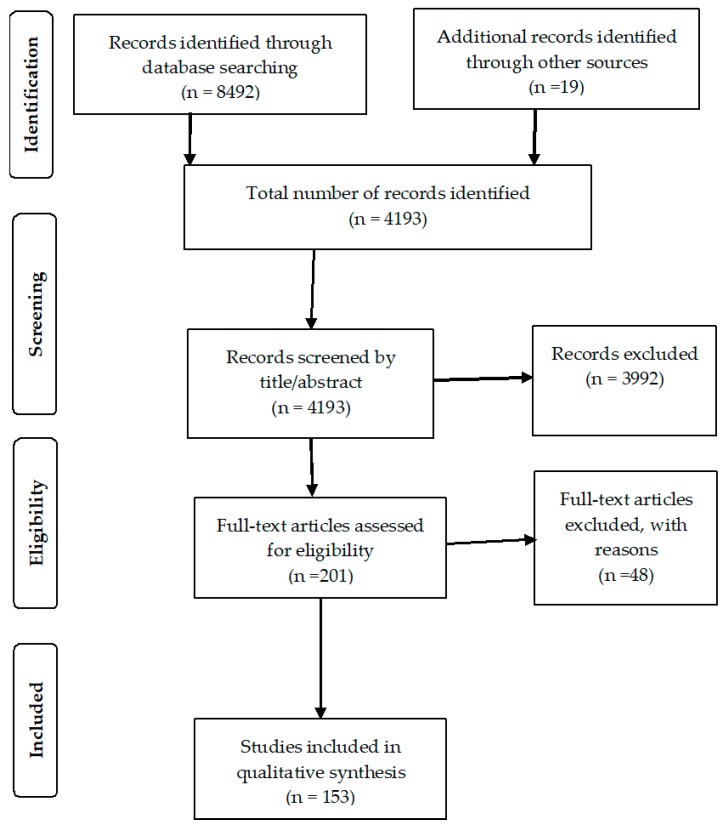
PRISMA flow diagram of the article selection process.

A total of 4193 unique articles were identified after excluding 4318 duplicates. Out of the remaining articles, 3992 were excluded because their abstracts and titles did not meet the eligibility requirements (Figure 1). From a full-text review of the remaining 201 articles a total of 153 were identified for the final review. The reasons for excluding 48 studies were that they had used non-spatial statistical methods (29 articles) or local or regional health survey data (18 articles), while one article was a systematic review (1 article).

### 3.2. Spatial Methods Used

In the set of articles chosen for review, the spatial methods that were used for disease mapping are shown in Table 1. Spatial smoothing and predictions were frequently employed (n = 108) and of which 32 and 76 articles made use of geostatistical data modelling and lattice data modelling, respectively. Spatial description aggregation methods (n = 25) and statistical spatial autocorrelation or clustering (n = 19) were the next most used spatial analysis methods. 

Most of the articles included in this review used data from DHS (n = 93). Country-specific surveys (n = 23), MIS (n = 17), MICS) (n = 5), AIDS Indictor Surveys (n = 4) were used in the other papers, and 11 articles used data from multiple surveys. All these surveys used multistage sampling designs that encamps stratification, cluster sampling, and unequal selection probabilities. These three complex sample design considerations have implications for statistical analyses of the survey data. There were 37 multicounty studies and country-specific articles, Malawi and Nigerian each contributed 17 studies, followed by South Africa with 11 studies, then Kenya with 10 studies.

### 3.3. Spatial Autocorrelation/Clustering

Nineteen (19) studies used at least one spatial autocorrelation or clustering technique to assess non-random spatial patterns and quantify correlation of spatial observations (Table 1). Kulldorff’s spatial scan statistics (n = 7), and Getis-Ord GI* statistic (n = 7) were most frequently used, followed by Global Moran’s *I*, Local Moran’s *I* and Anselin Local Moran’s I that were each used in three studies. K-function (n = 1) was also used (Table 2).

### 3.4. Spatial Modelling and Prediction

Of the 153 studies included in this review, most—138(90.1%)—used a standard or routine application of spatial methods. These involved studies that used spatial analysis methods embedded in GIS or spatial statistics software to measure spatial clustering and cluster detection and perform spatial modelling and predictions. Numerous studies (122 articles) used spatial modelling to describe relationships between the spatial health data and contextual factors to model and predict health data in space (Table 1 and Table 2). Out of these 122 studies, 76 (62.3%) concentrated on lattice data modelling, while 32 (26.2%) dealt with geostatistical data modelling. Almost all lattice and geostatistical analyses were implemented using Bayesian statistics. Only 15 studies endeavored to perform the spatial analysis using nonstandard methods (including joint spatial models and model assessment) or accounted for the survey design. Regarding spatial statistics software packages, BayesX was commonly used (n = 32) for modelling and prediction, followed by ArcGIS (n = 29), WINBUGS/OPENBUGS (n = 23), Integrated Laplace Approximation package (n = 16), and SaTSCAN (n = 9).

### 3.5. Spatial Methods Used

In this scoping review, several spatial statistical methods have been used in the extracted publications. These methods include descriptive spatial methods where features within a given area are simply summarized as totals or averages and then presented on that area (these are aggregation methods). These methods pose a challenge in the choice of the underlying population exposed, which may be problematic in SSA where data on population totals could be inadequate. Several forms of identifying specific observations or areas exhibiting spatial autocorrelation or clustering with their neighbors have been identified in the extracted articles. The spatial autocorrelation statistics methods employed included classic global statistics, such as Moran’s *I*, Geary’s *C* and Getis’s *G* [168,169], which estimate the overall degree of spatial autocorrelation in a dataset. They test for the presence and absence of non-random spatial patterns across the whole studied geographic area. On the other hand, local spatial autocorrelation analysis (also known as hotspot analysis) provides estimates disaggregated to the level of the spatial analysis units to identify local regions of strong autocorrelation. These are often identified by equivalent local spatial autocorrelation measures of Moran’s *I*, Geary’s *C* and Getis’s *G*. However, the most commonly used hotspot analysis is based on Anselin’s local indicator of spatial association (LISA) [168] and Kulldorff’s spatial scan statistic [170].

The widely used spatial statistics methods are the spatial regression (e.g., spatial lag in observed data and error terms, and geographically weighted regression (GWR)), spatial smoothing, and spatial interpolation, often employed by spatial epidemiologists to improve the estimation of health outcomes and burden. These methods have tools for deriving spatial surfaces from sampled data points or to smooth across polygons to create more robust estimates. Spatial interpolation or spatial prediction methods incorporate geographic information and values at a network of observed locations to estimate values at unobserved locations. In the traditional spatial analysis, the main spatial interpolation techniques include inverse distance weighting (IDW), Kriging, spline interpolation, and interpolating polynomials [171,172]. However, as the evidence shows, Bayesian spatial hierarchical modelling is becoming more effective than the conventional classical spatial analysis method, thanks to advanced computing power and Markov chain Monte Carlo (MCMC) methods [173]. They are now routinely being applied to model complex spatial relationships in large and multiple datasets using Bayesian statistical packages, which are freely available [10,11,12]. Most of the applications of disease mapping have been based on modelling lattice and “geostatistical” data. The former uses the so-called convolution model of Besag, York and Mollie (BYM) [174] and the latter uses the distance-based geostatistical model as expounded Diggle et al. [175].

### 3.6. Health Discipline and Themes

Before reviewing the articles included in this review, a list of research topics reflecting major health problems or themes in the SSA region was drawn. Eight major research themes were identified (Table 3). Some publications included at least two public health themes. Malaria or fever were predominately studied (n = 47), followed by health services/interventions coverage (n = 38), HIV/AIDS (n = 24), and mortality (n = 21).

### 3.7. Demography

More than half (54.9%) of the articles focused on populations aged less than 15 years, about 34.6% were aged above or equal to 15 years and 10.5% of the articles included all age groups (Table 1). We found limited literature items focusing on public health issues concerning males (<1%) and females (15%) exclusively, as most articles (84.3%) did not differentiate between the genders.

## 4. Discussion

This scoping review has demonstrated a variety of applications of spatial analysis techniques to household and health survey data in the SSA region. Spatial smoothing and prediction using Bayesian spatial statistics were predominantly used. Spatial autocorrelation and cluster detection were mostly fitted using frequentist methods and routines in GIS software. The most frequently studied health disciplines were malaria and fever followed by health services coverage and HIV/AIDS and health-related to mother and child health.

Despite the wide application of spatial methods in SSA, studies that only concentrated on men were scant (<1%). Additionally, there was a lack of studies concentrating on health program evaluation, possibly because data in this field might be sparse or not well captured in nationally representative health surveys. Most studies failed to account for complex survey design and data insufficiency, possibly due to data inadequacy about non-response, defective sampling frames, and missing information in addition to adjustments for clustering to ensure data representativeness and unbiased inferences. Few studies have developed and applied spatial statistics methods accounting for health survey design, but these were for data outside of SSA [176,177,178,179]. There is a lack of systematic and rigorous interrogation of spatial statistics, survey data, and software despite the need for new spatial analysis methods for validation, diagnostics, and predictions. Thus, the utilization of rich survey data sets remains sub-optimal because optimal analyses of such data demand in-depth assessment and the process and design collection of this kind of data must first be further developed. Most have tended to base their study papers with a “data analysts” mindset, with a heavy reliance on the implementation of developed biostatistics techniques in the widely available statistical software. Seldom have the authors thought critically around the development and validation of methods relevant to the problem being investigated. There will be a need for biostatistical expertise in analytical and innovative research, as well as adaptive skills to manage, analyze, and generate the data needed, including the use of existing data, to inform policymakers and local health service implementers [180]. A lack of these biostatistical skills could adversely affect the extent to which analyses and formulation of locally relevant scientific questions have been undertaken [5].

### 4.1. Limitations

Though the review was conducted adhering to PRIMSA-ScR guidelines, the search strategy used strategy might have missed studies that focused on some countries in SSA because our research included the term SSA only. We excluded studies that analysed health survey data, but the surveys were not nationally representative. We also did not interrogate sufficiently the methods used and the resulting findings. Most of the studies failed to account for the complex sampling design, which could have influenced the findings and conclusions drawn because standard spatial analyses generally underestimate the estimated variance of spatial estimates. Indeed, blind usage of available packages may adversely affect the extent to which analyses follow PRIMSA-ScR guidelines, and our search strategy might have missed studies that deployed spatial analysis techniques because we excluded papers published in languages other than English. There might also have been a risk of publication bias, which we did not assess. This review also excluded published research work that used spatial analyses on sentinel surveillance data. For example, spatial autocorrelation and inverse distance-weighted interpolation were used in [171,172,173,174,175,176,177,178,179,180,181,182,183] when spatial statistics were used to analyze HIV data of pregnant women attending antennal clinics (not health surveys).

### 4.2. Strengths

To the best of our knowledge, this is the first review to provide the range and depth of published studies using spatial analysis techniques to analyze the rich data obtained in nationally representative health surveys conducted in the SSA region. It includes health disciplines, themes and demographic information covering almost 30 years (1990–2018). Our findings demonstrate a wide range of applications of spatial analysis techniques dominated by modelling and prediction approaches based on Bayesian geostatistical and lattice data modelling.

### 4.3. Recommendations

Sample survey software should be used, especially for estimation of population parameters, and for descriptive and analytical analyses. Under certain circumstances, standard statistical packages can be used to provide results approximately equal to the results obtained from sample survey software. However, recognition of prevailing circumstances and an awareness of the potential pitfalls of using standard statistical packages require detailed information about the characteristics of the survey dataset used (e.g., sampling plan, weighting scheme, intra-cluster correlation) as well as a knowledge of the formulas and default options in standard software packages for weighted analyses. In the end, it seems easier and less time consuming to use a sample survey software package throughout.

Advanced analytical, innovative, and adaptive skills in spatial statistics should be used to manage and analyze existing survey data to better inform policymakers and local health service implementers. Indeed, new spatial methods might need to be developed for applications. We recommend a wide range of implementation examples from big health data, data future science and health systems to monitor and evaluate health program impacts, which are not well understood at the local level. Gender-specific studies focusing on an assessment of health interventions need to be conducted in the SSA region to provide further insights and enable profoundly informed decisions to improve public health concerning new areas of direction and research in SSA. Other obstacles in the region include the financial costs to obtain new data, the prolonged time before data become available for public use due to slow publication and/or bureaucratic processes that hinder data access and use.

Rigorous and coherent quality assessment of survey data is highly important, including design and coverage of sampling. Survey comparisons were often made when sample sizes, item measurement and context varied across years and were at times substantially and not necessarily congruent with national population numbers. Also, age ranges of respondents for the same data items differed across surveys, or across years within a survey. More could have been gained in studies had attempted to tackle key issues including data quality, data and methods triangulation and validation. A challenging, but potentially very fruitful undertaking could come from integrating household surveys with data from routine health information gathering, monitoring and surveillance systems. A focused agenda is recommended for data triangulation and contestability via linkage and validation studies that would allow drawing on complementary properties of different sources, assist in completeness estimations and improve our understanding of the accuracy attribution in the phenomena being studied. Such improved understanding holds clear gains for improved small area estimates, enhanced resource and service distribution, and, eventually, better meeting the health needs of the population.

Refinements of spatial methods and mapping levels are needed, e.g., by updating accessibility layers to include more recent and detailed road networks and settlement layers. This could also involve modelling key driving factors of the phenomena under study, such as poverty or access to sanitation, and then using these as covariates themselves. The effect that a country-specific focus, tailored as much as possible to a specific indicator, can have on mapping accuracies rather than using globally consistent covariates should be explored. Also, many socio-economic factors, not captured by the suite of covariates used, and often available at aggregate levels such as administrative units, could be obtained and their ability to improve mapping accuracy tested. The rising international focus on inequalities in the SDG-era requires a detailed and strong evidence base with an explicit quantification of uncertainties. Some studies provided sufficiently accurate prediction at an administrative unit that is relevant for policymaking and the allocation of resources. However, none of the studies looked at the issue of the Modifiable Areal Unit Problem (MAUP) in spatial analyses where an analysis based on a grouping unit may accidentally misrepresent or overstate actual risk variations [184]. Even if the data are grouped at the same level for analysis, the way the grouping scheme is used for spatial analysis may accidentally lead to misinterpretation of the spatial patterns. We recommend that studies consider, as part of sensitivity analysis, changing boundaries of levels to assess changes to the overall spatial patterns in the estimated phenomena.

Finally, we have already discussed at length how non-response, missing data, and self-reporting of health conditions pose statistical challenges when estimating small area spatial health variation. Missing data reduces the representativeness of the sample and can, therefore, distort the spatial inferences about a health measure. Perhaps a major feature of these survey data is their representativeness at national and regional levels, but not at the lower geographic level, which may not have been systemically covered sufficiently. Reliable estimates are highly associated with the number of observations falling into these lower levels. Conducting surveys that could generate representative data at the desired geographic level would be highly costly (due to an increase in sample sizes). Others have recommended choosing an appropriate spatial model after performing a systematic evaluation and validation of several spatial models for generating small area estimates [3,4,5,6]. Yet others have been novel by developing and validating non-standard spatial models, for example, those based on multivariate spatial models to model multiple health phenomena [95,105,153,154].

## 5. Conclusions

Comparisons and assessments of public health interventions and control programs at the sub-national level based on health survey data should consider survey design aspects when undertaking spatial analyses. Additionally, future research should focus on developing and evaluating spatial methods that leverage survey data in providing local estimates of health burdens. Several recommendations are made in this scoping review but most of them require strong skills and analytic capacity. Thus, further expansion and strengthening of analytic capacity in the development and application of spatial analysis methods relating to health survey data constitute the main message of our critical and overarching recommendation.

## Figures and Tables

**Table 1:** Classification of the articles selected for review (n = 153).

Focus of the Publication	Number	Percentage	Reference
*Spatial Analysis Method*			
Description or Aggregation methods	25	16.3%	[4,18,19,20,21,22,23,24,25,26,27,28,29,30,31,32,33,34,35,36,37,38,39,40,41]
Autocorrelation/Clustering	19	12.4%	[42,43,44,45,46,47,48,49,50,51,52,53,54,55,56,57,58,59,60]
*Spatial Regression and Interpolation*			
Kriging	8	5.2%	[61,62,63,64,65,66,67,68]
Inverse Distance Weighting	1	0.7%	[69]
Weighted Kernel Regression	1	0.7%	[70]
Geographically Weighted Regression (GWR)	4	2.6%	[71,72,73,74]
*Spatial Smoothing and Prediction*			
Geostatistical data modelling	32	20.9%	[6,41,61,62,64,65,66,67,68,72,75,76,77,78,79,80,81,82,83,84,85,86,87,88,89,90,91,92,93,94,95,96]
Lattice data modelling	76	49.7%	[5,6,69,70,71,74,97,98,99,100,101,102,103,104,105,106,107,108,109,110,111,112,113,114,115,116,117,118,119,120,121,122,123,124,125,126,127,128,129,130,131,132,133,134,135,136,137,138,139,140,141,142,143,144,145,146,147,148,149,150,151,152,153,154,155,156,157,158,159,160,161,162,163,164,165,166,167]
*Application Techniques*			
Nonstandard applications (e.g., spatial analysis model assessment, joint spatial modelling, accounting for survey design)	15	9.8%	[5,6,74,122,123,128,132,142,150,151,153,155,157,159,160]
*Survey design and inadequacy*			
Survey design	4	2.6%	[74,122,153,160]
Non-response/missing	2	1.3%	[122,159]
*Computer Software Package*			
BayesX	32	20.9%	[88,108,110,113,114,115,116,117,118,119,120,123,124,127,129,131,133,135,138,140,142,143,144,145,147,148,149,150,154,156,161,167]
WINBUGS/OPENBUGS	23	15.0%	[47,61,65,67,75,80,91,92,104,112,119,123,128,130,134,142,146,147,155,159,160,161,165]
ArcGIS	29	19.9%	[22,23,28,31,39,42,43,44,45,46,48,49,51,53,54,57,58,71,72,73,75,76,98,101,103,105,106,107,109]
R-prev package	3	1.3%	[5,6,102]
QGIS	1	0.7%	[20]
GeoDA	4	2.6%	[21,43,59,71]
SaTSCAN	9	5.9%	[45,48,50,51,52,54,103,108,109]
R-survey and mgcv package	1	0.7%	[34]
ArcView	1	0.7%	[36]
MapInfo professional	2	1.3%	[47,104]
GeoR	1	0.7%	[59]
INLA	16	10.4%	[4,46,74,82,89,93,94,95,122,126,132,134,136,139,162,163]
Own code: Fortran	4	3.0%	[63,64,68,78]
*Study Population*			
*Age group*			
Children (<15 years old)	82	53.6%	[19,21,25,27,29,31,33,35,37,40,42,43,44,45,54,55,56,57,60,61,62,64,65,66,67,68,70,71,75,76,79,81,84,86,87,89,91,92,94,95,98,101,104,106,110,111,112,113,115,117,118,119,120,121,122,125,126,127,131,132,133,134,135,136,137,138,139,140,141,142,143,144,145,146,154,155,156,157,161,162,163,167]
Adults (≥15 years old)	50	32.7%	[4,5,6,18,22,23,28,30,32,34,36,39,43,46,47,48,49,50,52,58,63,65,69,74,83,85,96,97,99,100,102,105,107,108,109,114,116,123,124,128,129,147,148,149,150,151,153,158,159,160]
All age groups	17	11.1%	[20,24,26,38,53,72,77,80,82,88,90,103,130,152,165,166,167]
*Gender*			
Male	1	0.7%	[34]
Female	23	15%	[18,23,28,32,36,46,58,59,74,88,96,99,100,107,109,114,116,124,128,148,150,154,160]
Both genders	125	81.6%	[4,5,6,19,20,21,22,24,25,26,27,29,30,31,33,35,37,38,39,40,41,42,43,44,45,47,48,49,50,51,52,53,54,55,56,57,60,62,63,64,65,66,67,68,69,70,71,72,73,75,76,77,78,79,81,82,83,84,85,86,87,89,90,91,92,93,94,95,97,98,101,102,103,104,105,106,108,110,111,112,113,115,117,118,119,120,121,122,123,125,126,127,129,130,131,132,133,134,135,136,137,138,139,140,141,142,143,144,145,146,149,151,152,153,155,156,157,158,159,161,162,163,164,165,166,167]
*Health Surveys*			
Demographic Health Survey	93	60.8%	[4,5,18,20,21,22,23,25,26,27,28,30,33,36,38,39,40,42,44,45,48,49,50,52,54,55,56,57,58,59,60,61,62,68,69,71,76,81,82,85,86,87,88,95,97,98,99,100,102,103,105,106,109,110,111,113,115,116,118,120,121,122,123,124,127,129,131,133,135,137,140,141,142,143,144,146,147,148,150,151,153,154,155,156,157,158,159,162,163,167]
Malaria Indicator Survey	17	11.1%	[31,37,60,63,64,67,70,77,78,79,80,89,92,93,101,107,136]
Multiple Indicator Cluster Survey	5	3.3	[75,113,125,140,145]
AIDS Indicator survey	4	2.6%	[74,153,160,166]
Multi-Surveys	12	7.8%	[6,20,24,53,66,90,96,108,119,130,159,165]
Country-Specific Surveys	23	15.0%	[29,32,43,46,47,51,65,72,73,82,83,91,96,104,112,126,128,132,134,139,149,152,164]
*Country of Study*			
Angola	1	0.7%	[78]
Burkina Faso	3	2%	[88,89,134]
Cameroon	2	1.3%	[103,119]
Democratic Republic of Congo	9	6.5%	[30,38,42,69,98,114,120,127,138]
Ethiopia	7	4.6%	[45,50,55,58,105,109,167]
Equatorial Guinea	1	0.7%	[51]
Egypt	1	0.7%	[140]
Ghana	2	1.3%	[4,29]
Kenya	10	6.5%	[20,31,34,46,72,74,90,122,153,160]
Lesotho	2	1.3%	[22,97]
Madagascar	1	0.7%	[136]
Malawi	17	11.1%	[77,102,110,113,114,120,121,123,133,134,145,154,156,157,158,161,162]
Mali	1	0.7%	[68]
Mozambique	2	1.3%	[32,43]
Multi-Country	37	24.2%	[5,6,10,19,20,21,22,24,25,26,28,33,37,39,40,44,48,49,53,54,60,61,62,66,71,76,83,84,86,87,91,95,96,119,144,163,165]
Namibia	2	1.3%	[101,155]
Nigeria	17	11.1%	[32,46,77,83,87,97,111,114,125,126,130,134,136,138,139,143,144]
Rwanda	3	2.0%	[97,100,116]
Senegal	2	1.3%	[81,82]
Somalia	5	3.3%	[75,126,132,139,164]
South Africa	11	6.5%	[47,52,73,104,123,128,146,147,150,151,152]
Sudan	1	0.7%	[130]
Tanzania	4	2.6%	[63,70,106,166]
Uganda	6	3.9%	[56,57,65,67,92,108]
Zambia	5	3.3%	[80,93,96,107,129]
Zimbabwe	2	1.3%	[36,119]

INLA: Integrated Nested Laplace Approximations.

**Table 2:** Main spatial analysis techniques used in data analysis.

Method Category	Method	No. of References	Reference
Spatial Clustering and regression	Global Moran’s *I*	3	[42,44,45]
	Local Moran’s *I* (LISA)	3	[30,46,47]
	Kulldorff’s spatial scan statistic	7	[45,48,49,50,52,53,54]
	Getis-Ord GI* statistic	7	[43,45,51,55,56,57,58]
	Anselin Local Moran’s *I*	3	[44,45,59]
	K-function	1	[60]
	Spatial Prediction and Interpolation	10	[61,62,63,64,65,66,67,68,69,70]
	Generalized Weighted Regression	4	[71,72,73,74]
Spatial modelling and prediction	Bayesian geostatistical models	32	[6,41,61,62,64,65,66,67,68,72,75,76,77,78,79,80,81,82,83,84,85,86,87,88,89,90,91,92,93,94,95,96]
	Bayesian conditional autoregressive (CAR) models	76	[5,6,69,70,71,74,97,98,99,100,101,102,103,104,105,106,107,108,109,110,111,112,113,114,115,116,117,118,119,120,121,122,123,124,125,126,127,128,129,130,131,132,133,134,135,136,137,138,139,140,141,142,143,144,145,146,147,148,149,150,151,152,153,154,155,156,157,158,159,160,161,162,163,164,165,166,167]
	Joint modelling	12	[5,74,126,128,132,142,150,151,155,157,159,160]

**Table 3:** Application areas of spatial methods.

Health Discipline	Frequency
Mortality	21
Malaria and fever	47
HIV/AIDS	24
Non-communicable diseases	9
Malnutrition	12
Diarrhoea	7
Health services coverage	38
Other *	5

* birth intervals; sexual debut; schistosomiasis; pneumonia.

## Supplementary Materials

The following are available online at https://www.mdpi.com/1660-4601/17/9/3070/s1, Table S1: Checklist for Preferred Reporting Items for Systematic reviews and Meta-Analyses extension for Scoping Reviews (PRISMA-ScR) Checklist.
